# Deep Learning for Microfluidic-Assisted *Caenorhabditis elegans* Multi-Parameter Identification Using YOLOv7

**DOI:** 10.3390/mi14071339

**Published:** 2023-06-29

**Authors:** Jie Zhang, Shuhe Liu, Hang Yuan, Ruiqi Yong, Sixuan Duan, Yifan Li, Joseph Spencer, Eng Gee Lim, Limin Yu, Pengfei Song

**Affiliations:** 1 School of Advanced Technology, Xi’an Jiaotong-Liverpool University, Suzhou 215123, China; 2 Department of Electrical and Electronic Engineering, University of Liverpool, Liverpool L693BX, UK

**Keywords:** deep learning, *C. elegans* sorting, object detection, YOLOv7, multi-parameter sorting

## Abstract

The *Caenorhabditis elegans (C. elegans)* is an ideal model organism for studying human diseases and genetics due to its transparency and suitability for optical imaging. However, manually sorting a large population of *C. elegans* for experiments is tedious and inefficient. The microfluidic-assisted *C. elegans* sorting chip is considered a promising platform to address this issue due to its automation and ease of operation. Nevertheless, automated *C. elegans* sorting with multiple parameters requires efficient identification technology due to the different research demands for worm phenotypes. To improve the efficiency and accuracy of multi-parameter sorting, we developed a deep learning model using You Only Look Once (YOLO)v7 to detect and recognize *C. elegans* automatically. We used a dataset of 3931 annotated worms in microfluidic chips from various studies. Our model showed higher precision in automated *C. elegans* identification than YOLOv5 and Faster R-CNN, achieving a mean average precision (mAP) at a 0.5 intersection over a union (mAP@0.5) threshold of 99.56%. Additionally, our model demonstrated good generalization ability, achieving an mAP@0.5 of 94.21% on an external validation set. Our model can efficiently and accurately identify and calculate multiple phenotypes of worms, including size, movement speed, and fluorescence. The multi-parameter identification model can improve sorting efficiency and potentially promote the development of automated and integrated microfluidic platforms.

## 1. Introduction

Since the 1970s, when *Caenorhabditis elegans (C. elegans)* was discovered by Sydney Brenner, it has been recognized as an excellent model organism for studying the genetic regulation of organ development and programmed cell death [1,2,3]. *C. elegans* plays a valuable role in studying genetics, drug development, and cell biology due to its short developmental cycle (3 to 4 days), small size (1 to 1.3 mm), ease of cultivation, and high homology (~65%) of its genetic pathways with humans [4,5,6]. The phenotypes of *C. elegans* differ (e.g., size and movement speed) at different developmental stages, meeting various specific research demands [7,8].

The size, motility, and fluorescence expression of *C. elegans* are promising research subjects in specific fields [9]. The varied sizes of *C. elegans* can facilitate an understanding of complex biological processes, including embryogenesis, development, disease, and aging [10,11]. The motility of worms is closely linked to the genetic mechanisms for maintaining balance and movement, such as muscle contraction and synaptic plasticity [12,13,14]. Otherwise, the fluorescence expression of worms is a useful tool for labeling and observing. For example, green fluorescent protein (GFP) is widely used for optical in vivo imaging and phenotypic observation [11,15].

Efficient and accurate *C. elegans* sorting has always been a significant challenge in related studies [16]. *C. elegans* can be sorted based on different phenotypes, such as fluorescence expression, morphology, and motility characteristics [17]. The traditional *C. elegans* sorting method requires manual selection under a microscope, which is labor-intensive and requires skilled operators [18,19,20]. Later, the complex object parametric analyzer and sorter (COPAS) was developed for efficient and high-throughput worm sorting, utilizing fluorescent signals and other optical features [21,22]. However, the widespread use of COPAS is limited due to its bulkiness, high cost, and operational complexity [23]. In recent years, the advancements of lab-on-a-chip (microfluidics) have made it a promising platform for facilitating various studies, such as microinjection and drug screening [24]. Microfluidic chips are well suited for *C. elegans* studies due to their highly matched dimensions (in the hundreds of microns to a few millimeters) [25,26,27,28]. In addition, they have the advantages of low cost, good biocompatibility, and easy operation [29,30]. Thanks to software-controlled microscopy and various programmable devices such as micropumps and microvalves, microfluidic chips can rapidly and accurately sort worms [28]. However, these chips still have limitations in integrated, high-content, and multi-parameter sorting [31]. Achieving multi-parameter sorting integrated on a single microfluidic chip is challenging.

Computer vision can automate experimental processes to improve efficiency, enabling a fast response time and customization for multiple complex backgrounds [32,33,34,35,36]. Multiple computer vision techniques have shown good potential for the automated detection of *C. elegans* [37,38,39,40,41]. Based on deep learning algorithms, a real-time detector system has previously been developed for accurate worm localization, classification, and profile prediction, including the Mask region-based convolutional neural network (R-CNN), Faster R-CNN, and You Only Look Once (YOLO)v5 [41]. The latest version of YOLOv7 has been recognized as one of the most accurate and fastest real-time object detectors, making it ideal for optimizing applications in *C. elegans.* [42,43,44]. However, applications combining YOLOv7 with microfluidics are yet to be developed.

**Figure 1:**
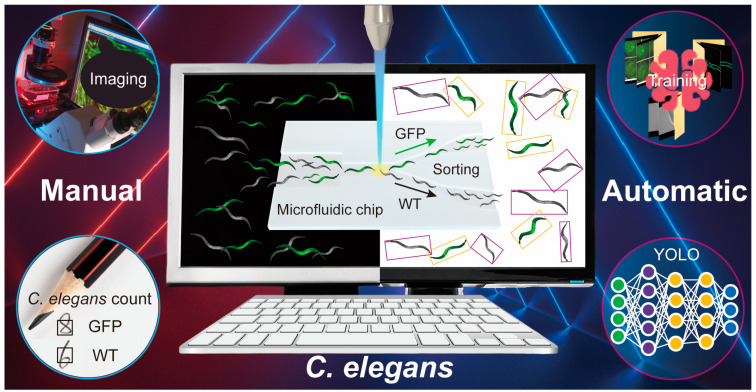
Schematic of deep learning for microfluidic-assisted *C. elegans* multi-parameter identification using YOLOv7.

In this study, we reported a deep learning model using YOLOv7 for microfluidic-assisted *C. elegans* multi-parameter identification (Figure 1). We extracted *C. elegans* images in microfluidic chips from various studies as our datasets. Then, we annotated 3931 *C. elegans* to provide the data required for YOLOv7 training. These datasets, with a large number of annotated images, ensure the effectiveness of our deep learning model. Otherwise, we compared the performance of YOLOv7 to YOLOv5 and Faster R-CNN in detecting *C. elegans* in microfluidic chips. Furthermore, we examined the generalizability of YOLOv7 using an external validation set. The size and movement speed of *C. elegans* can be computationally identified by YOLOv7, enabling the classification of multiple phenotypes (size, motility, and fluorescence). Our model achieved a high precision with a mean average precision (mAP) at a 0.5 intersection over a union (mAP@0.5) threshold of 99.56%. Our model also exhibited good generalization abilities when tested on an external validation set, achieving a mAP@0.5 of 94.21%. Thanks to the identification of multiple parameters with our model, the efficiency of microfluidic-assisted *C. elegans* research has been improved. Through this functional integration developed by our study, the automation and integration of microfluidic systems are expected to be further improved.

## 2. Methods

This section discusses the methods used in the following sections: Section 2.1, application scenario; Section 2.2, image acquisition; Section 2.3, dataset preparation; Section 2.4, model configuration and installation; Section 2.5, evaluation metrics; Section 2.6, training configuration; Section 2.7, comparison of YOLOv7, YOLOv5, and Faster R-CNN parameters; and Section 2.8, calculation of worm size and movement speed.

### 2.1. Application Scenario

Microfluidic chips can be combined well with optical microscopes (e.g., inverted microscopes and fluorescence microscopes) in biology labs for *C. elegans* research. The combination can potentially improve the efficiency and precision of biological research but requires the assistance of computer algorithms. Thus, our model focuses on the application scenario of microfluidic chips. To capture images, perform morphological and physiological measurements, and sort individual *C. elegans*, we can combine the model with our previously developed microfluidic chip, which is capable of sequentially loading *C. elegans* one-by-one (success rate~90.3%) [28]. Our model can detect and identify images of worms moving in microfluidic devices captured by high-speed cameras of microscopes. With image feedback, the model can accurately calculate and determine the phenotype information of each *C. elegans* (e.g., size, movement speed, and fluorescence) and actively sort the required *C. elegans* from a mixture of *C. elegans* with different phenotypes. Our real-time vision-based multi-parameter identification model can improve the automation level of microfluidic systems.

### 2.2. Image Acquisition

The dataset was obtained from published studies that utilized microfluidic chips for *C. elegans* sorting. The videos from published studies were downloaded as raw data [45,46,47,48,49,50,51,52,53,54,55,56,57,58,59,60,61,62,63,64]. To improve the model’s robustness and the data’s diversity, we created datasets using reference videos with different imaging conditions from various papers. The acquired videos were sampled at 10-frame intervals to extract cropped worm images and annotate them, resulting in 3931 annotation boxes. After the training phase, the acquired data were sorted into training, testing, and validation sets based on the GFP and WT classification.

### 2.3. Dataset Preparation

A large amount of annotated data are needed when training the deep learning model. In this study, the worms in each image were annotated manually using the image annotation software LabelImg, which supports the Visual Object Classes (VOC) format [65,66]. This software can generate Extensible Markup Language (XML) files for the model.

To improve the performance of the model training process, we selected *C. elegans*, which was easily recognizable in the channels as the primary training object. To promote the convergence performance of the model, we carefully selected several worms that were not in the channel as part of the dataset.

**Figure 2:**
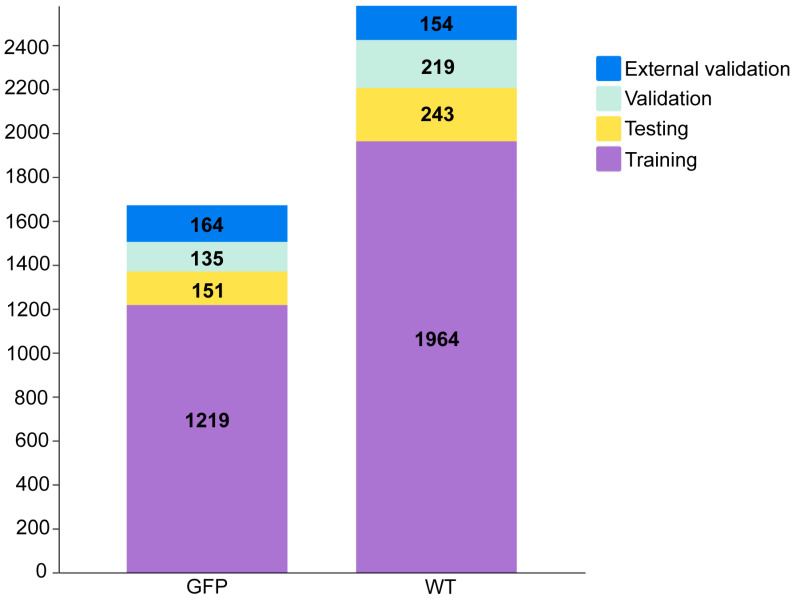
Numbers of annotated GFP and WT in the training, testing, validation, and external validation sets.

Each worm is located within an annotation box, and the number of these boxes determines the dataset size in Figure 2. There were 2426 annotation boxes for the WT category and 1505 for the GFP category. The annotation boxes were categorized into four sets, namely training, testing, validation, and external validation sets. The training and testing sets were used for model training and internal evaluation, while the validation set was utilized for model adjustment. To verify the generalization ability of the model, we also selected 318 annotated worms from separated video sources as the external validation set. The split data ratio was set to 9:1 for training and validation sets to the testing set. The training set to the validation set ratio was also set to 9:1. According to the above ratio, the annotated GFP and WT numbers in each set are shown in Figure 2.

### 2.4. Model Configuration and Installation

**Figure 3:**
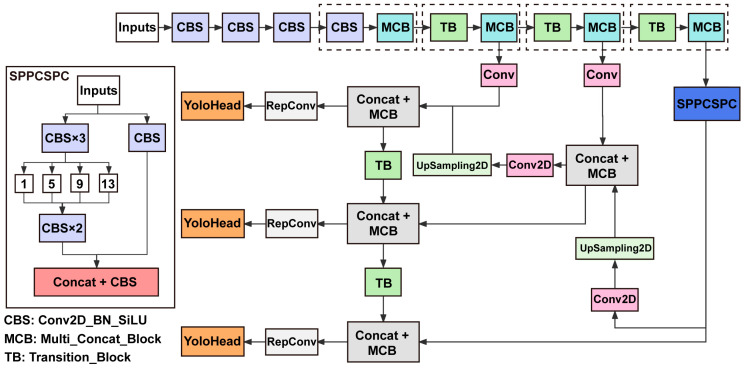
The overall structure is generic YOLO architecture. The backbone extracts the feature from the image. The neck enhances the feature map by combining different scales. The head provides the bounding box for detection. SPPCSPC, spatial pyramid pooling convolutional set pooling convolutional; Conv, convolution; Conv2D, two-dimensional convolutional layer; RepConv, reparameterized convolution; UpSampling2D, two-dimensional up-sampling layer; Concat, concatenate; CBS, Conv2D_BN_SiLU (convolutional standardization activation function); MCB, Multi_Concat_Block (multi-branch stacking module); TB, Transition_Block (transition block).

Our model had an overall structure, as shown in Figure 3. The spatial pyramid pooling convolutional set pooling convolutional (SPPCSPC) module is used in object detection algorithms, specifically in the YOLOv7 model. The SPPCSPC module refers to a Cross Stage Partial Network (CSPNet) with a spatial pyramid pooling (SPP) block. The SPP block pools the input tensor with various kernel sizes and concatenates the results. It shows the difference in maximum pooling. Different pooling amounts correspond to different targets. Moreover, the SPP block has four different scales of maximum pooling (the resolutions of 1 × 1, 5 × 5, 9 × 9, and 13 × 13) with four kinds of perceptual fields, distinguishing large and small targets.

The innovative transition block (TB) is used for down-sampling. In convolutional neural networks, two commonly used transition modules for down-sampling are a convolutional layer with a kernel size of 3 × 3 and a stride of 2 × 2, or a max pooling layer with a stride of 2 × 2. YOLOv7 combines these two transition modules into a TB with two branches. The left branch of the TB is a 2 × 2 stride max pooling followed by a 1 × 1 convolutional layer. The right branch of the TB is a 1 × 1 convolutional layer followed by a convolutional layer with a kernel size of 3 × 3 and a stride of 2 × 2. The results from both branches are stacked together when outputting.

**Figure 4:**
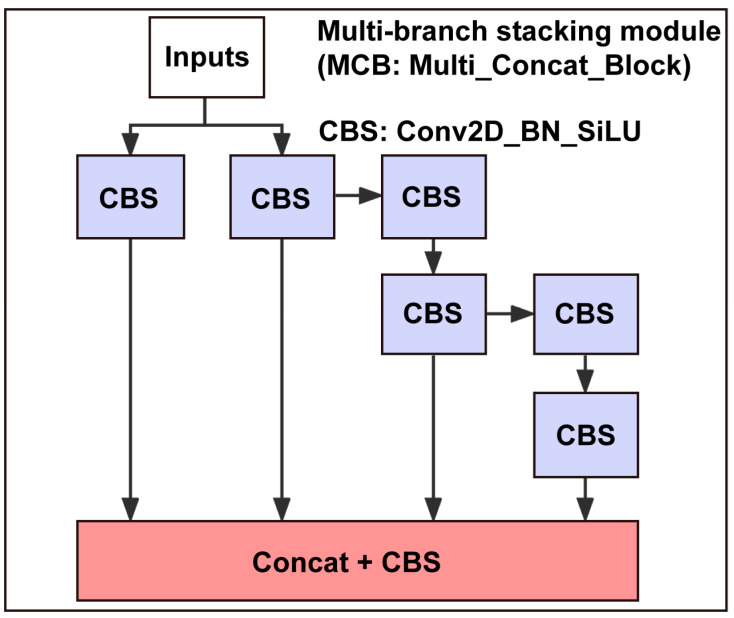
Multi-branch stacking module (i.e., MCB of Figure 3). The module is designed to enhance the accuracy of multi-target detection. During training, a module is split into multiple branches of the same or different modules. Then, multiple branches are integrated into a single module by designing multi-scale feature extraction. Finally, a multi-branch stacking module is added to further improve the precision of object detection.

The overall structure of our model utilized multi-branch stacking modules, represented by the MCB in Figure 3 and illustrated in Figure 4. In this module, the input of the final stacking module contained multiple branches. From left to right, the four branches shown in Figure 4 consist of 1, 1, 3, and 5 CBS, respectively. After these branches were stacked, they conducted another CBS for feature integration. The dense stacking corresponded to a dense residual structure that included internal residual blocks. While increasing the depth of the neural network can optimize and improve model accuracy, it may lead to vanishing gradient problems. Internal residual blocks with skip connections can mitigate this issue.

YOLOv7 constructs a feature pyramid network (FPN) to enhance the feature extraction. The FPN is located between convolution (Conv) and reparameterized convolution (RepConv) structures in Figure 3, including Conv but excluding RepConv. In the feature utilization stage, YOLOv7 extracts three feature layers at different positions in the backbone for object detection: the middle, lower middle, and bottom layers [67]. Each feature point on each feature layer in YOLOv7 has three prior boxes. The channel number of each feature layer can be adjusted using convolution so that the final number of channels is proportional to the number of distinct categories. These three layers have different shapes when the input is (640, 640, 3): feat = (80, 80, 512), feat2 = (40, 40, 1024), and feat3 = (20, 20, 1024). The FPN is constructed using these three feature layers, combing feature layers of various shapes to obtain better features. In YOLOv7, the FPN produces three enhanced features with shapes of (20, 20, 512), (40, 40, 256), and (80, 80, 128), which are passed to the YOLO detection head for result prediction. Unlike previous YOLO models, YOLOv7 employs a RepConv structure before the YOLO detection head. RepConv structure introduces new residual structures uniquely designed to aid in training. During prediction, these structures can be effectively equivalent to a standard 3 × 3 convolution. Furthermore, they can reduce network complexity while maintaining prediction performance.

### 2.5. Evaluation Metrics

The performance of YOLOv7 was evaluated using several metrics. Recall is the ratio of all true positives (objects correctly identified) to the total of true positives and false negatives (objects missed). Precision is the ratio of true positives to the total of true and false positives (objects misidentified). AP is the area under the precision–recall curve, and its average value for each class is the mAP. The intersection over union (IoU) threshold was used to measure the degree of overlap between the predicted bounding box and the actual ground truth box. It is defined mathematically as the ratio of the intersection area of the two boxes to their union. The F1 score, the harmonic mean of precision, and recall were used to evaluate the overall performance of the model. Moreover, the detection speed of the model in frames per second (FPS) on the tracked video was evaluated. Finally, the IoU threshold for the tracked frame was set to 0.5.

The training process for this model involved two stages: the frozen stage and the unfrozen stage. During the frozen stage, the backbone was frozen and the feature extraction network remained unchanged. The training was conducted for 50 epochs. The backbone was unfrozen and the feature extraction network was modified during the unfrozen stage. The training was conducted for 300 epochs. The learning rate of the model was dynamically adjusted, with a range of 1 × 10^−4^ to 1 × 10^−2^. The stochastic gradient descent optimizer was used with a weight decay of 5 × 10^−4^. Real-time training progress monitoring was achieved by performing an average evaluation every 10 epochs, which provided valuable information on the model performance. This information helped to identify any overfitting or underfitting issues and enabled the adjustment of hyperparameters or the training process to improve model performance. For example, if the precision of the model decreases, reducing the learning rate or increasing the number of epochs may help. Alternatively, if the model is overfitting, regularization techniques may be applied to prevent the memorization of training data. By conducting regular evaluations, the training process can be optimized for maximum model performance.

The YOLO detection head generates prediction results of three feature layers with shapes of (N, 20, 20, 255), (N, 40, 40, 255), and (N, 80, 80, 255), respectively. However, these predictions do not correspond to the positions of the final predicted boxes on the image. They need to be decoded to complete the process. In YOLOv7, each feature point on each feature layer has three anchor boxes. The final 255 values of each feature layer can be split into three sets of 85 parameters, which correspond to the 3 anchor boxes. These 85 parameters can be reshaped into (N, 20, 20, 3, 85), (N, 40, 40, 3, 85), and (N, 80, 80, 3, 85) for the three feature layers, respectively. Among these parameters, 85 can be further divided into 4 + 1 + 80. The first 4 parameters are used to adjust the regression parameters of each feature point and obtain the predicted box. The 5th parameter determines whether each feature point contains an object. The last 80 parameters identify the object type in each feature point.

First, the feature layer divides the image into grid cells in the object detection model. Then, any grid cell that falls within an object’s corresponding box is used to predict the object. Next, score filtering and non-maximum suppression (NMS) filtering are performed to reduce the number of prediction boxes. Score filtering selects boxes with scores that meet the confidence threshold, thus significantly reducing the number of boxes to be processed during the overlap removal. Meanwhile, NMS selects the box with the highest score for a certain category within a certain region. To apply NMS to each category independently, each category is looped through, and the boxes with lower scores for the same category are filtered out. After filtering, the boxes for each category are sorted in descending order of their scores. Then, the box with the highest score is removed each time, and its overlap with all other predicted boxes is calculated. Boxes with high overlap are removed. Finally, the remaining prediction boxes are used for the final predictions. In our case, we used a score filtering value of 0.5 and an NMS parameter (nms_iou) of 0.3.

### 2.6. Training Configuration

We trained our model on the Windows 10 operating system using an NVIDIA GeForce RTX 3090 GPU with 32 GB RAM. We implemented our model using version 1.7.1 of the PyTorch machine learning framework. Moreover, our model depended on NVIDIA’s CUDA (version 11.1) and CUDNN libraries for support.

### 2.7. Comparison of the YOLOv7, YOLOv5, and Faster R-CNN Parameters

Our study employed YOLOv7 to identify the WT and GFP datasets and compare its performance against YOLOv5 and Faster R-CNN. We have used the best hyperparameters for three models. The input size of the images for both YOLOv7 and YOLOv5 was 640 × 640, while the size for Faster R-CNN was 600 × 600. Additionally, the batch size for training three models was the same and equaled 3. Table 1 summarizes the parameters used to train the three models.

### 2.8. Calculation of Worm Size and Movement Speed

To obtain the size of the *C. elegans*, we determined the actual size of each pixel point based on the scale, and then computed the pixel positions of the worm’s boundaries. The number of occupied pixels was used to determine the worm size. To determine the movement speed of the *C. elegans*, we recorded the Greenwich time and left boundary of the frame for each detected image, and calculated the difference in time and boundary position between adjacent frames. We conducted tests using two distinct microfluidic chips selected from the dataset videos. Then, we converted pixel numbers into actual lengths of worms based on microfluidic chip lengths of 4.6875 × 10^3^ μm (chip 1) and 3.4286 × 10^3^ μm (chip 2), and resolutions of 863 × 725 pixels (chip 1) and 318 × 232 pixels (chip 2). Consequently, we obtained the worm size and movement speed.

## 3. Results

**Figure 5:**
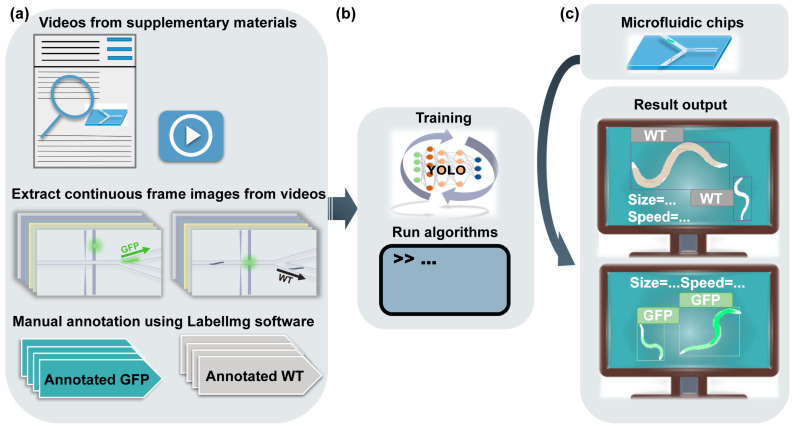
The training procedure and the general process of using YOLOv7 to detect *C. elegans* during sorting in microfluidic chips. (**a**) Videos from published microfluidic-assisted sorting studies were searched and captured as images, which were annotated using LabelImg software. (**b**) These annotated images were used in the training process of YOLOv7. (**c**) The model detected WT and GFP *C. elegans* and synthesized the final recognition output size and speed.

We performed the process shown in Figure 5 using YOLOv7 to detect *C. elegans* during sorting in microfluidic chips. We compared the performance of YOLOv7, YOLOv5, and Faster R-CNN in Table 2. YOLOv7 achieved the highest mAP of 99.56% for detecting the WT and GFP datasets, outperforming YOLOv5 and Faster R-CNN.

We compared the performance of YOLOv7, YOLOv5, and Faster R-CNN in *C. elegans* detection, and the results of the evaluation metrics are shown in Table 3. The specific results of the testing set are presented in Figures S1–S9 of the Supplementary Materials. The three models used the same number of images for object detection, with 2426 and 1505 images for WT and GFP, respectively. YOLOv7 showed the fastest detection speed among the three models, with an FPS of 52.112. Moreover, YOLOv7 exhibited higher precision, achieving 100% and 97.39% for detecting WT and GFP, respectively. Although Faster R-CNN showed a slightly better recall of 99.35% in GFP detection than the other two models, overall, YOLOv7 demonstrated higher F1 scores in both WT and GFP detection.

An external validation set can help ensure our model can generalize to new data rather than overfitting the training data. Therefore, we compared the performance of YOLOv7, YOLOv5, and Faster R-CNN on an external validation set to evaluate their generalization ability. The results of the evaluation metrics are shown in Table 4. The specific results of the external validation set are presented in Figures S10–S18 of the Supplementary Materials. The three models used the same number of annotation boxes from the external validation set, with 154 and 164 annotated WT and GFP *C. elegans*, respectively. YOLOv7 showed the fastest detection speed among the three models, with an FPS of 52.508. In addition, YOLOv7 exhibited the highest precision for both WT and GFP, reaching 95.24% and 89.47%, respectively. YOLOv7 also showed the highest mAP of 94.21%, indicating that our model has robust detection and classification capabilities for *C. elegans*. Otherwise, YOLOv7 had the highest recall of 89.47% for GFP, although its recall for WT was slightly lower than that of Faster R-CNN but still better than that of YOLOv5. Overall, YOLOv7 has a better balance between precision and recall, with F1 scores of 0.95 and 0.89 for WT and GFP, respectively.

**Figure 6:**
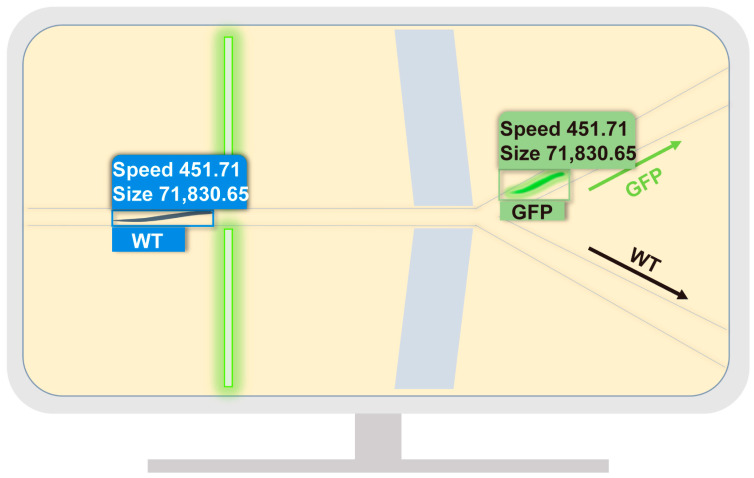
The trained model detected WT and GFP *C. elegans* in input images and output the recognized size and speed.

We applied our model to recognize *C. elegans* automatically in microfluidic chips from videos. The mixed populations of WT and GFP *C. elegans* were imaged in the microfluidic chips under an optical microscope. Our model could identify the fluorescent phenotype of these worms (WT or GFP) and calculate their size and speed (Figure 6). In practical application scenarios, microfluidic chips can be combined with our model to sort GFP and WT worms into different channels to obtain a single target population. Moreover, combined with different microfluidic chip designs, the size and speed output results can also serve as sorting criteria. As shown in Table 5, we employed twenty-eight worms for identification in our model, including twenty-four WT and four GFP worms from chip 1 (Worm1–11 and Worm25–28) and chip 2 (Worm12–24). The largest sized worm was 94,394.17 μm^2^ and the smallest sized was 11,547.36 μm^2^. The fastest worm had a speed of 2827.30 μm/s, while the slowest was 46.13 μm/s. Since the recognition was performed on videos, the speed 0 is invalid due to interference from the video frame rate. Variations in *C. elegans*’ movement speed may arise due to differences in video frame rates, air pump settings, and other experimental conditions of the microfluidic chips [68]. However, it is important to note that these factors do not invalidate the obtained results. On the contrary, they showcase the adaptability of our model to diverse microfluidic application environments, highlighting the robustness and effectiveness of our approach.

## 4. Discussion

Researchers are increasingly using object detectors to study *C. elegans*. While fluorescent techniques are helpful for tracking and sorting *C. elegans*, sorting typically involves multiple parameters. Therefore, combining deep learning models with microfluidic chips is a critical way to achieve platform automation and improve the efficiency of multi-parameter identification. We developed a target detection model to identify WT and GFP *C. elegans* in microfluidic chips and generate their size and speed information. This approach can improve the precision and efficiency of microfluidic chips during *C. elegans* sorting, reducing errors associated with manual counting.

We extracted *C. elegans* in microfluidic chips from videos and annotated them to create a dataset. We trained three commonly used detectors (YOLOv7, YOLOv5, and Faster R-CNN) using the dataset and validated their performance using an external validation set. The comparison results of their performance showed that YOLOv7 outperformed the others in terms of recall, precision, and generalization ability. Therefore, we ultimately chose to apply YOLOv7 in our study. Our study provided a dataset of thousands of annotated worms. Additionally, we developed an engineering prototype that combines microfluidic chips and deep learning technology for efficient *C. elegans* sorting. The model provided a solution to identify multiple parameters of worms in microfluidic chips.

In microfluidic chips, worms are introduced into microchannels, where they experience fluid flow and confinement. These microfluidic channels are typically designed with specific geometries and dimensions to create controlled flow conditions [17]. The worms are not completely immobilized but rather constrained to move and navigate within the available space, interacting with the fluid and channel walls. Some microfluidic chips are also equipped with active forces, such as different types of pumps. The assistance of these active forces helps ensure efficient sorting and prevent any potential interference or blockage within the chips. The applied active forces and specific channel geometries influence worm behaviors, allowing them to be sorted into different bifurcated channels based on predefined criteria such as size and speed. The movement of worms may exhibit inherent randomness and a tendency to interact with the channel sidewalls, displaying exploratory behavior [68]. In further research, integrating our algorithm and microfluidic chips is expected to measure, control, and predict worm behaviors precisely within microchannels, contributing to a more reliable analysis.

We can also easily implement our identification model for efficient and automated multi-parameter *C. elegans* sorting with our developed *C. elegans* soring microfluidic systems, which are capable of continuously loading *C. elegans* with a 90.3% success rate [28]. Based on the current performance of our model, we anticipate achieving a high recognition success rate and target population purity in practical applications. For example, *C. elegans* is commonly used in drug screening and exhibits a wide range of variations in movement. These variations can arise from various factors such as individual differences, drug effects, and environmental conditions [69]. Addressing the variations in worm movement is crucial for accurately assessing the effects of drugs in experiments. By leveraging our model, the efficient and automated analysis of worm locomotion under different drug treatments can be performed, thereby accelerating the drug screening process and enhancing screening accuracy. Furthermore, precise measurements of worm movement variations can be used to evaluate the toxicological effects of drugs and provide robust support for drug safety assessments. We will further explore the potential applications of our deep learning model in these areas.

## 5. Conclusions

*C. elegans* is a model organism with great potential in human disease and genetics research. The efficient and accurate sorting of *C. elegans* has always been a challenge in related research. Microfluidic chips are promising for *C. elegans* sorting due to their low cost, good biocompatibility, and simple operation, compared with the cumbersome and inefficient manual operation and expensive COPAS. However, automated *C. elegans* sorting with multiple parameters requires efficient identification technology due to the different research demands for worm phenotypes. Therefore, we extracted and annotated 3931 worms from videos as a dataset and selected YOLOv7 through the training, comparison, and external validation of three detectors (YOLOv7, YOLOv5, and Faster R-CNN). YOLOv7 showed high precision in worm recognition, achieving an mAP@0.5 of 99.56%. In addition, our model has a good generalization ability, achieving an mAP@0.5 of 94.21% on the external validation set while efficiently and accurately identifying multiple *C. elegans* phenotypes, including size, movement speed, and fluorescence. This study comprehensively evaluated the performance of YOLOv7 as a *C. elegans* recognition detector. Otherwise, we established a new engineering prototype that combines microfluidics and deep learning technology. We will further develop highly integrated and automated microfluidic chips based on our model to better serve *C. elegans*-related research.

## Figures and Tables

**Table 1:** Parameters used to train YOLOv7, YOLOv5, and Faster R-CNN.

Parameters	Values
Optimizers	stochastic gradient descent
Learning rate	1 × 10^−4^~1 × 10^−2^
Momentum	0.937
Learning decay	5 × 10^−4^
Pretrained	Microsoft Common Objects in Context dataset
Number of epochs	300

**Table 2:** Performance comparison between YOLOv7, YOLOv5, and Faster R-CNN.

Model	Backbone	Annotation Number	mAP (%)	AP of the WT (%)	AP of the GFP (%)
Faster R-CNN	Resnet50	3931	98.93	99.64	98.22
YOLOv5	Darknet-53	3931	99.03	100	98.06
YOLOv7	Darknet-53	3931	99.56	100	99.12

**Table 3:** Comparison of different models in target detection (YOLOv7, YOLOv5, and Faster R-CNN).

		Metrics							
Model	Sample Type	Detection Speed (FPS)	mAP (%)	Model Size	AP (%)	F1	Recall (%)	Precision (%)	Annotation Number
Faster R-CNN	WT	19.032	98.93	108	99.64	0.84	100	71.88	2426
	GFP				98.22	0.85	99.35	74.76	1505
YOLOv5	WT	32.146	99.03	27.1	100	1	100	100	2426
	GFP				98.06	0.96	96.13	95.51	1505
YOLOv7	WT	52.112	99.56	142	100	1	100	100	2426
	GFP				99.12	0.97	96.13	97.39	1505

**Table 4:** Comparison of external validation results among different models (YOLOv7, YOLOv5, and Faster R-CNN).

		Metrics							
Model	Sample Type	Detection Speed (FPS)	mAP (%)	Model Size	AP (%)	F1	Recall (%)	Precision (%)	Annotation Number
Faster R-CNN	WT	19.550	66.22	108	64.32	0.54	100	36.84	154
	GFP				68.12	0.56	78.95	42.86	164
YOLOv5	WT	32.434	88.83	27.1	94.72	0.73	90.48	61.29	154
	GFP				82.93	0.73	63.16	85.71	164
YOLOv7	WT	52.508	94.21	142	98.94	0.95	95.24	95.24	154
	GFP				89.47	0.89	89.47	89.47	164

**Table 5:** Identification of WT and GFP *C. elegans* size and speed.

Sample Type	Sample	Size (μm^2^)	Speed (μm/s)
WT	Worm1	25,310.88	0.00
WT	Worm2	23,094.72	0.00
WT	Worm3	71,830.65	451.71
WT	Worm4	13,989.51	61.05
WT	Worm5	16,067.16	0.00
WT	Worm6	22,249.08	511.05
WT	Worm7	16,823.92	970.29
WT	Worm8	12,188.88	0.00
WT	Worm9	11,547.36	0.00
WT	Worm10	34,372.35	1400.80
WT	Worm11	16,271.28	0.00
WT	Worm12	37,507.72	68.51
WT	Worm13	56,537.78	55.95
WT	Worm14	93,056.36	77.43
WT	Worm15	76,269.74	61.26
WT	Worm16	52,304.80	63.24
WT	Worm17	48,196.18	93.28
WT	Worm18	37,507.72	85.85
WT	Worm19	56,537.78	67.00
WT	Worm20	55,724.91	46.52
WT	Worm21	52,304.80	46.13
WT	Worm22	48,196.18	98.84
WT	Worm23	62,866.91	131.96
WT	Worm24	68,319.63	70.41
GFP	Worm25	40,444.92	0.00
GFP	Worm26	67,731.39	2827.30
GFP	Worm27	94,394.17	1216.61
GFP	Worm28	73,016.64	2028.55

## Data Availability

The code used and the trained models in this study are freely available and are hosted in a dedicated open-source repository on GitHub [70].

## Supplementary Materials

The following supporting information can be downloaded at: https://www.mdpi.com/article/10.3390/mi14071339/s1, Specific results of the testing set. Figure S1. (a) The mAP, (b) the log-average miss rate, and (c) the ground-truth results in Faster R-CNN. Figure S2. (a) The recall, (b) the F1 score, (c) the precision, and (d) the AP results of WT *C. elegans* in Faster R-CNN. Figure S3. (a) The recall, (b) the F1 score, (c) the precision, and (d) the AP results of GFP *C. elegans* in Faster R-CNN. Figure S4. (a) The mAP, (b) the log-average miss rate, and (c) the ground-truth results in YOLOv5. Figure S5. (a) The recall, (b) the F1 score, (c) the precision, and (d) the AP results of WT *C. elegans* in YOLOv5. Figure S6. (a) The recall, (b) the F1 score, (c) the precision, and (d) the AP results of GFP *C. elegans* in YOLOv5. Figure S7. (a) The mAP, (b) the log-average miss rate, and (c) the ground-truth results in YOLOv7. Figure S8. (a) The recall, (b) the F1 score, (c) the precision, and (d) the AP results of WT *C. elegans* in YOLOv7. Figure S9. (a) The recall, (b) the F1 score, (c) the precision, and (d) the AP results of GFP *C. elegans* in YOLOv7. Specific results of the external validation set. Figure S10. (a) The mAP, (b) the log-average miss rate, and (c) the ground-truth results in Faster R-CNN. Figure S11. (a) The recall, (b) the F1 score, (c) the precision, and (d) the AP results of WT *C. elegans* in Faster R-CNN. Figure S12. (a) The recall, (b) the F1 score, (c) the precision, and (d) the AP results of GFP *C. elegans* in Faster R-CNN. Figure S13. (a) The mAP, (b) the log-average miss rate, and (c) the ground-truth results in YOLOv5. Figure S14. (a) The recall, (b) the F1 score, (c) the precision, and (d) the AP results of WT *C. elegans* in YOLOv5. Figure S15. (a) The recall, (b) the F1 score, (c) precision, and (d) the AP results of GFP *C. elegans* in YOLOv5. Figure S16. (a) The mAP, (b) the log-average miss rate, and (c) the ground-truth results in YOLOv7. Figure S17. (a) The recall, (b) the F1 score, (c) the precision, and (d) the AP results of WT *C. elegans* in YOLOv7. Figure S18. (a) The recall, (b) the F1 score, (c) the precision, and (d) the AP results of GFP *C. elegans* in YOLOv7.
