# Supplementary material for: Deep Learning for Microfluidic-Assisted Caenorhabditis elegans Multi-Parameter Identification Using YOLOv7
Source: Micromachines (Basel). 2023 Jun 29;14(7):1339. doi: 10.3390/mi14071339 (PMC10386376; doi:10.3390/mi14071339)
Supplement: Supplementary file 1 [file micromachines-14-01339-s001.zip › micromachines-2369995-supplementary.pdf]

Specific Results of the Testing Set

1. Faster R-CNN

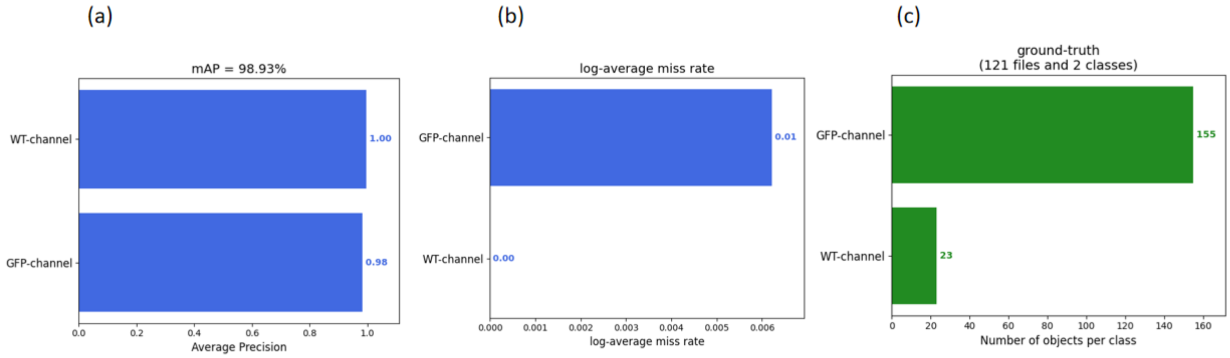

Figure S1. (a) The mAP, (b) the log-average miss rate, and (c) the ground-truth results in Faster R-CNN.

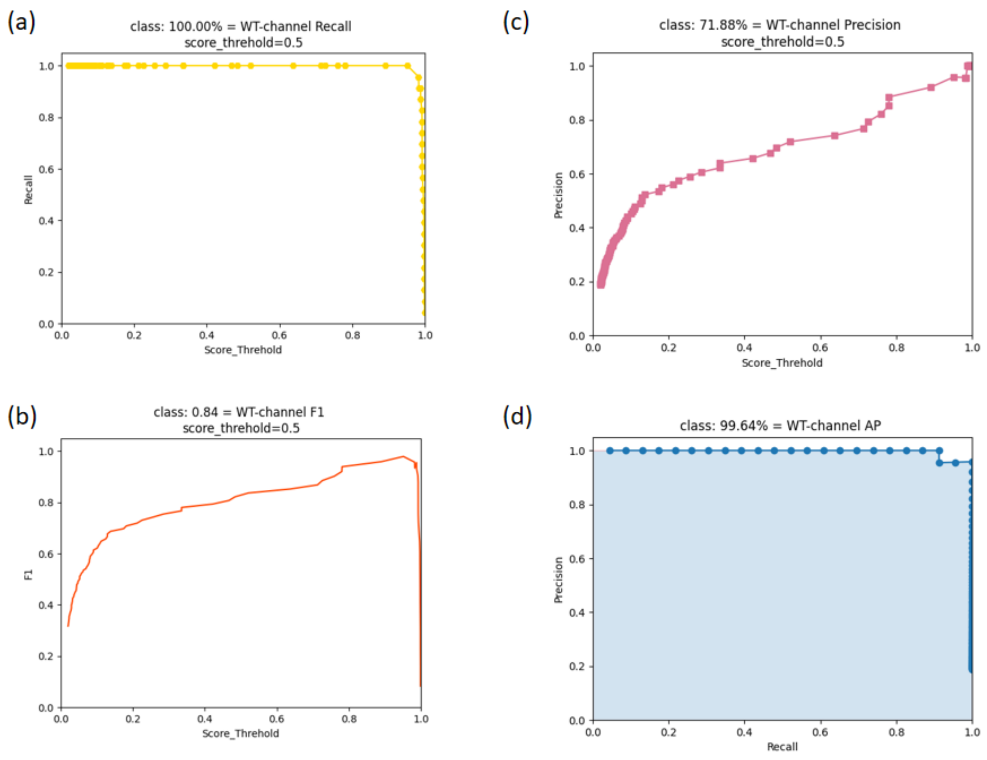

Figure S2. (a) The recall, (b) the F1 score, (c) the precision, and (d) the AP results of WT *C. elegans* detection in Faster R-CNN.

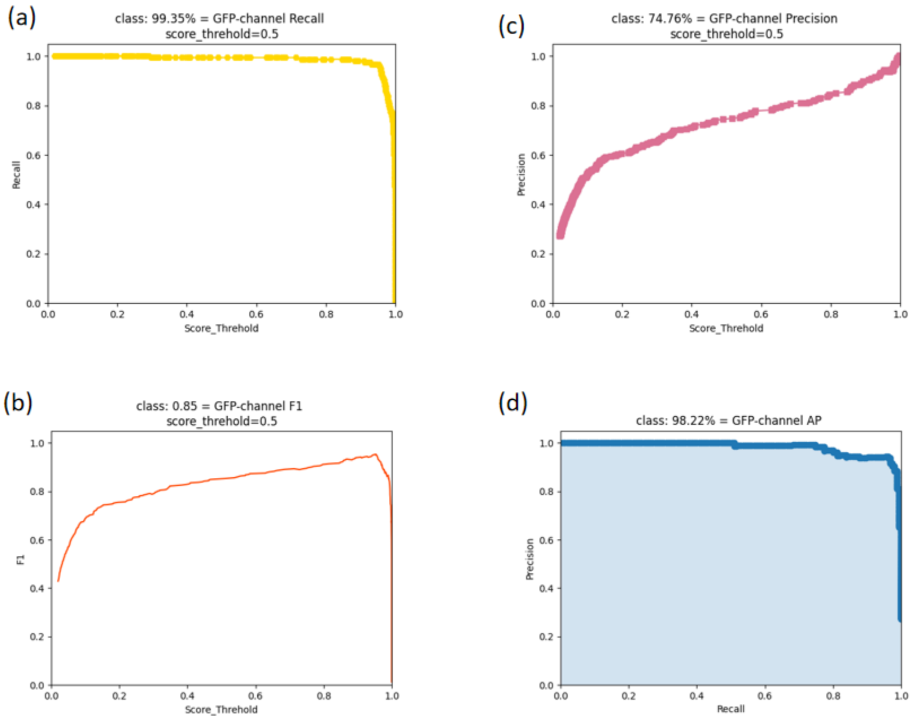

Figure S3. (a) The recall, (b) the F1 score, (c) the precision, and (d) the AP results of GFP *C. elegans* detection in Faster R-CNN.

2. YOLOv5

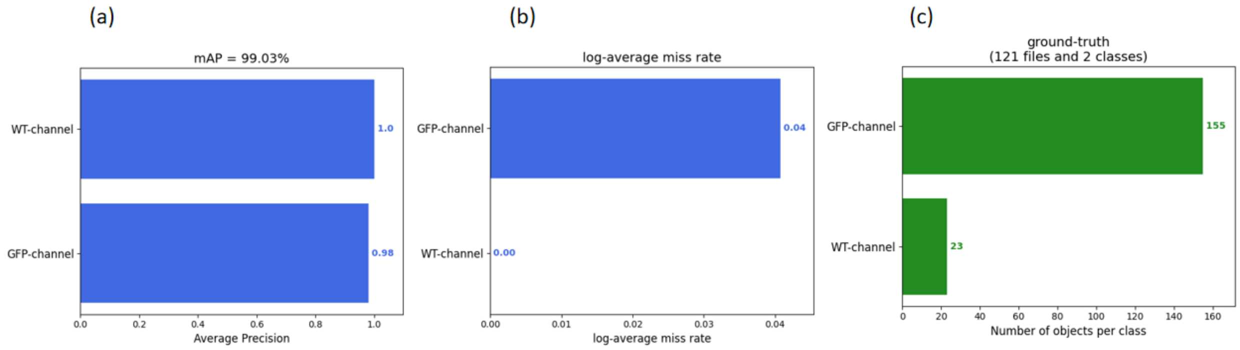

Figure S4. (a) The mAP, (b) the log-average miss rate, and (c) the ground-truth results in YOLOv5.

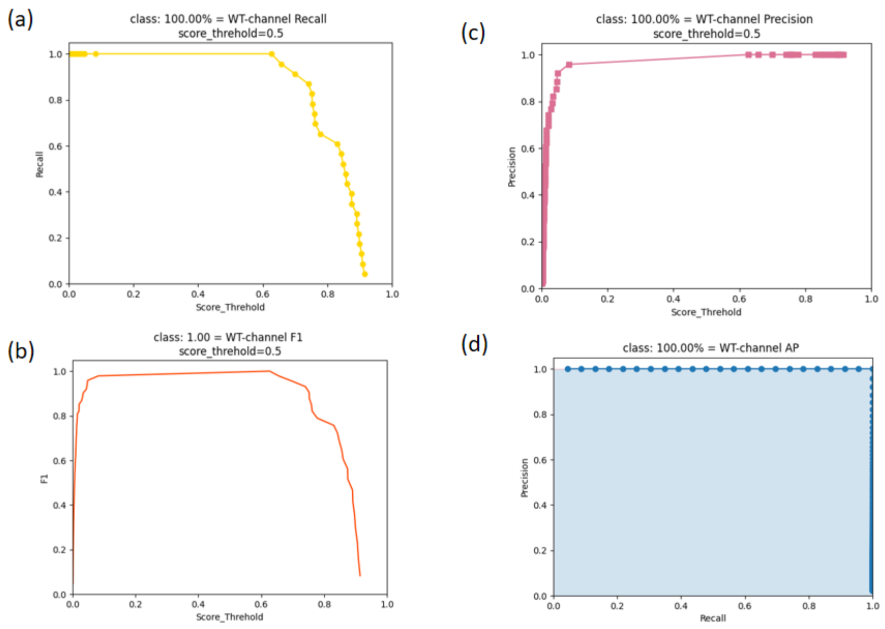

Figure S5. (a) The recall, (b) the F1 score, (c) the precision, and (d) the AP results of WT *C. elegans* detection in YOLOv5.

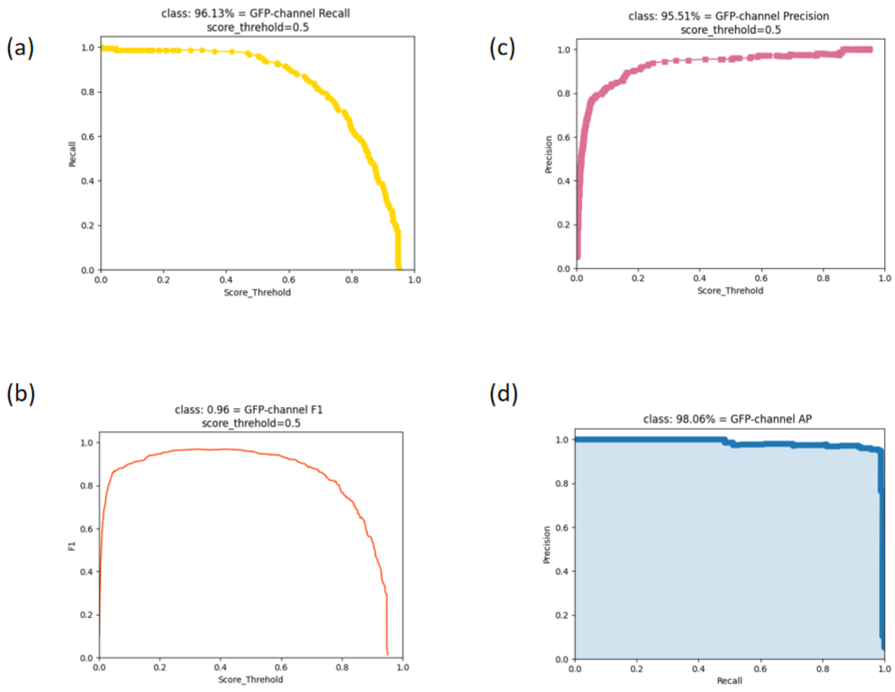

Figure S6. (a) The recall, (b) the F1 score, (c) the precision, and (d) the AP results of GFP *C. elegans* detection in YOLOv5.

3. YOLOv7

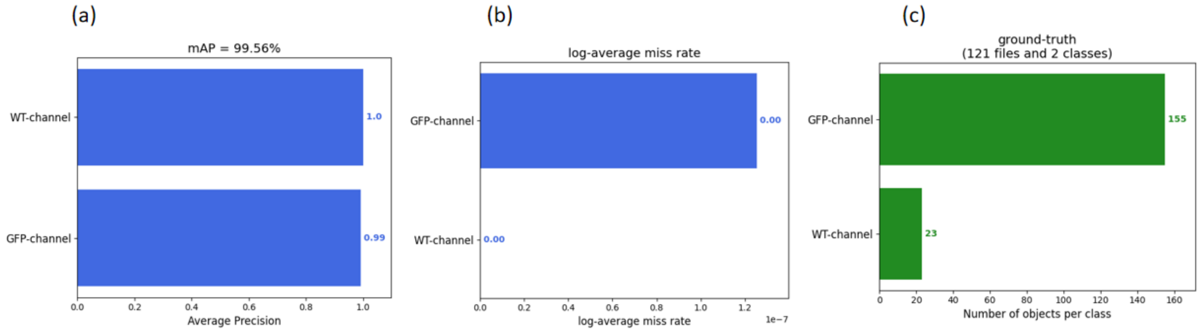

Figure S7. (a) The mAP, (b) the log-average miss rate, and (c) the ground-truth results in YOLOv7.

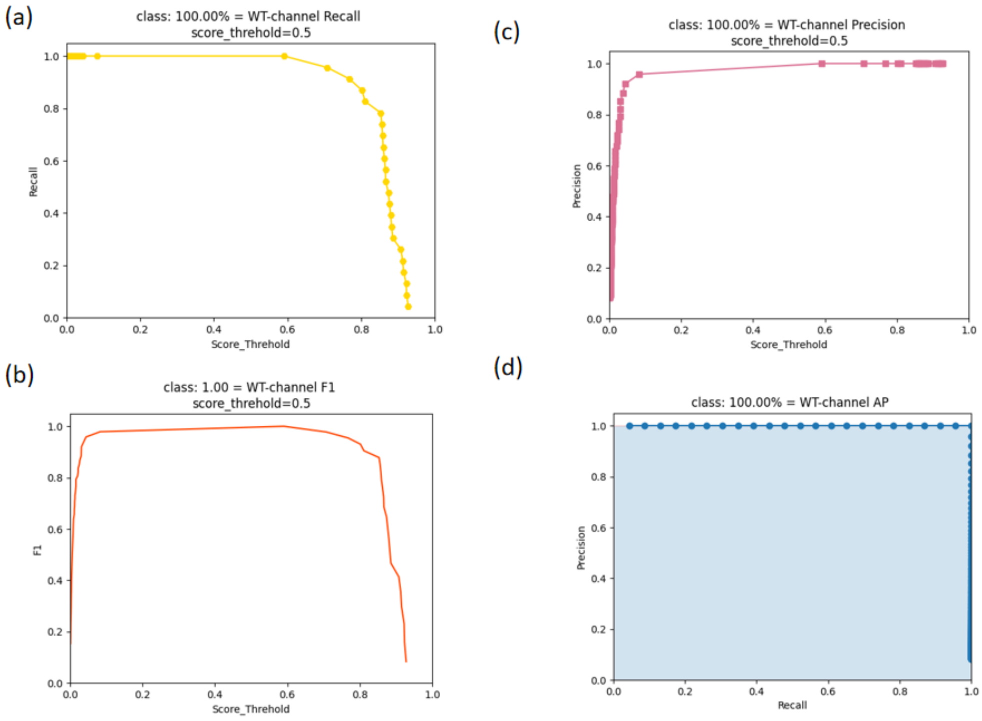

Figure S8. (a) The recall, (b) the F1 score, (c) the precision, and (d) the AP results of WT *C. elegans* detection in YOLOv7.

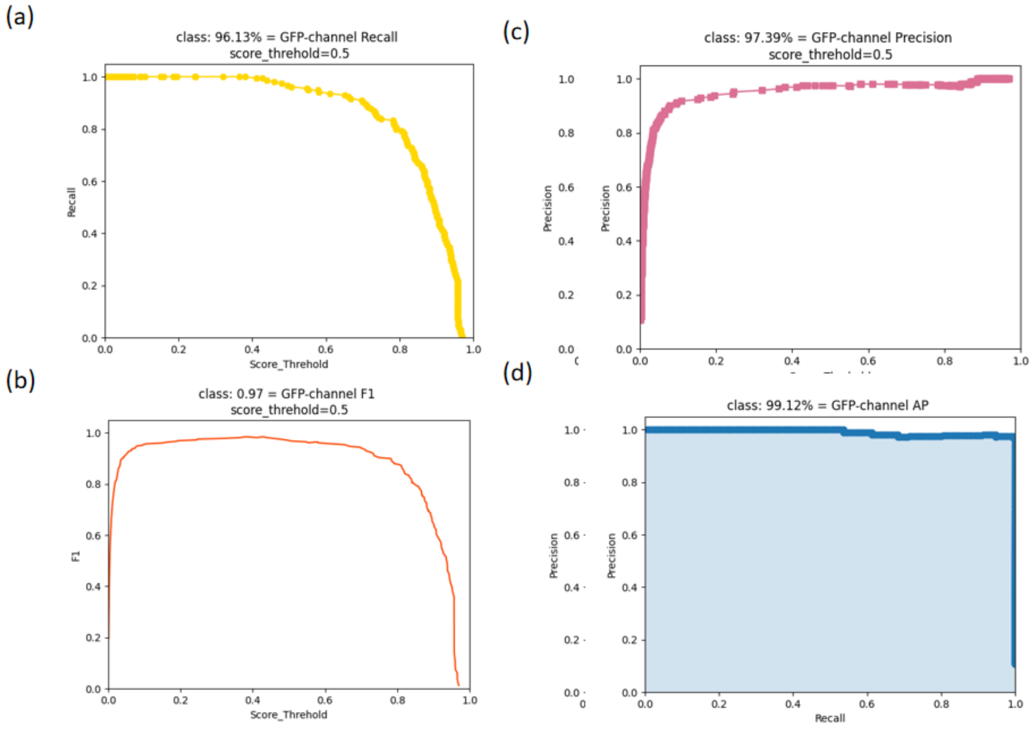

Figure S9. (a) The recall, (b) the F1 score, (c) the precision, and (d) the AP results of GFP *C. elegans* detection in YOLOv7.

Specific Results of the External Validation Set

4. Faster R-CNN

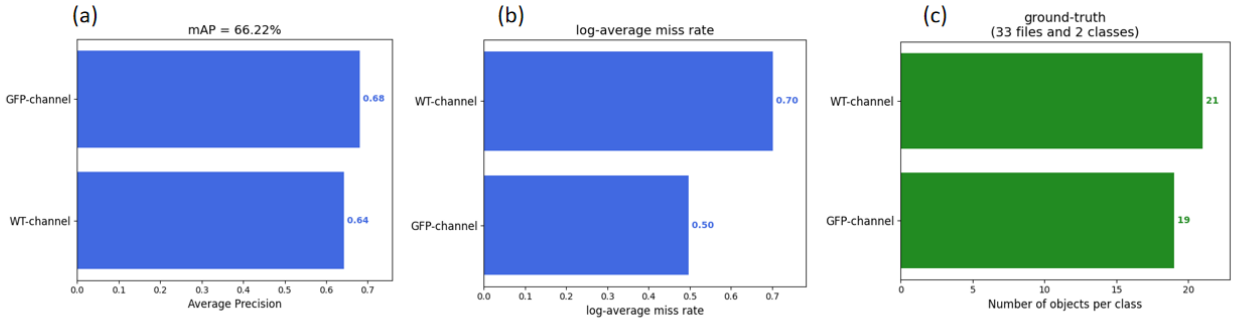

Figure S10. (a) The mAP, (b) the log-average miss rate, and (c) the ground-truth results in Faster R-CNN.

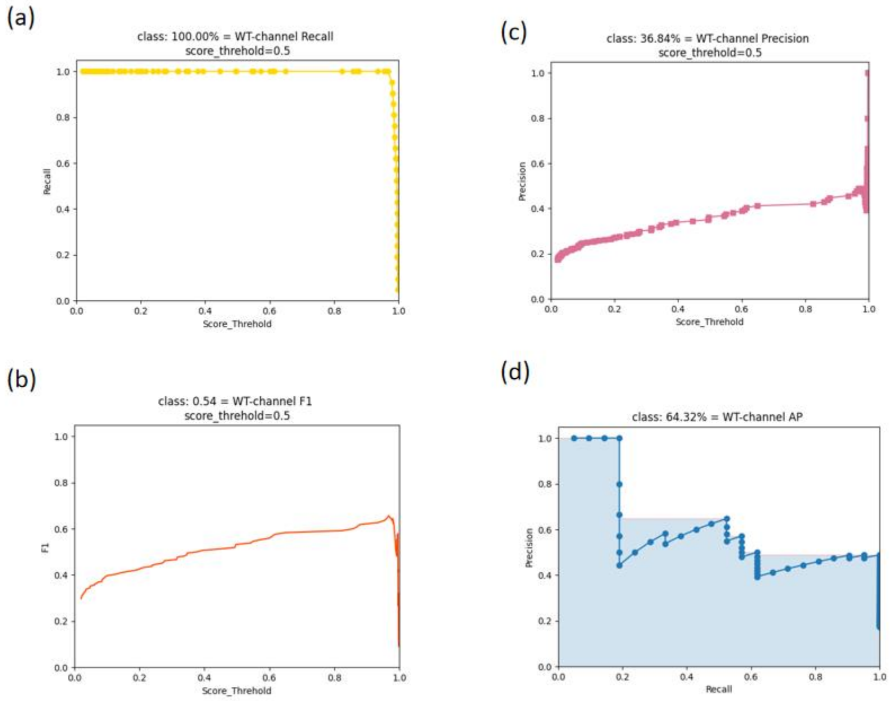

Figure S11. (a) The recall, (b) the F1 score, (c) the precision, and (d) the AP results of WT *C. elegans* detection in Faster R-CNN.

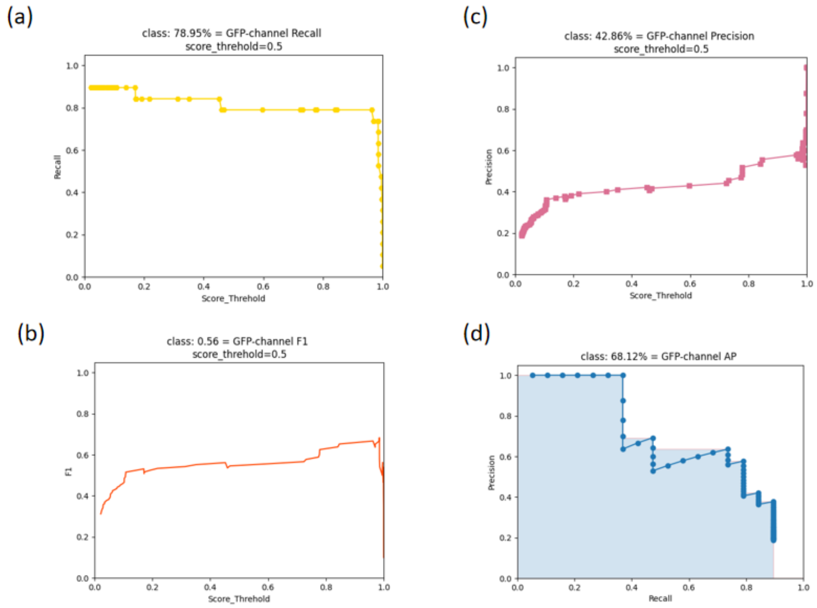

Figure S12. (a) The recall, (b) the F1 score, (c) the precision, and (d) the AP results of GFP *C. elegans* detection in Faster R-CNN.

5. YOLOv5

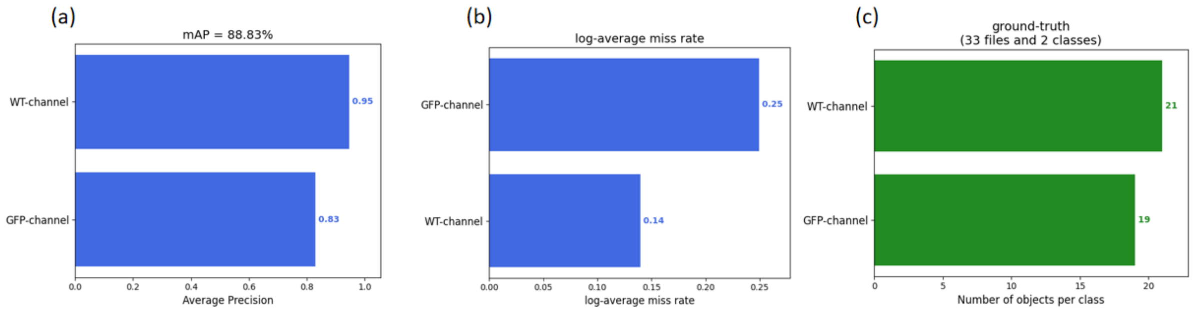

Figure S13. (a) The mAP, (b) the log-average miss rate, and (c) the ground-truth results in YOLOv5.

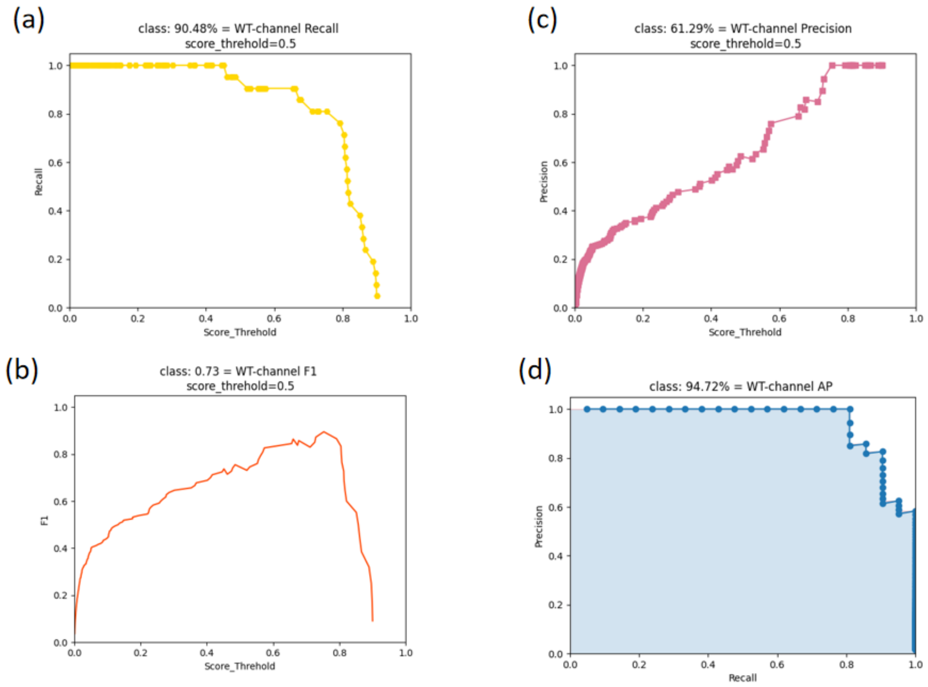

Figure S14. (a) The recall, (b) the F1 score, (c) the precision, and (d) the AP results of WT *C. elegans* detection in YOLOv5.

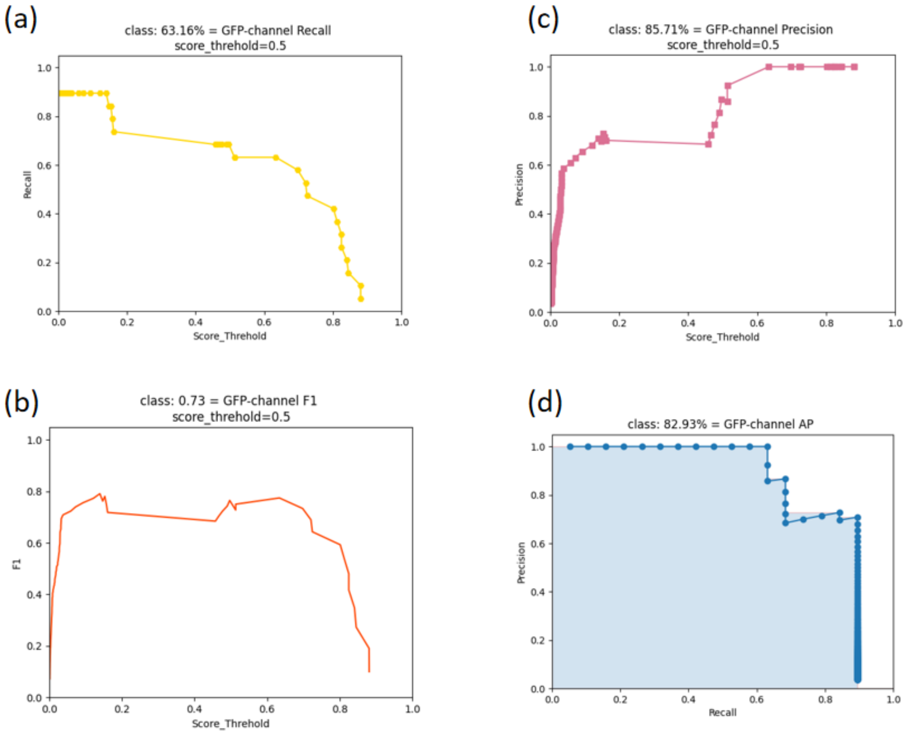

Figure S15. (a) The recall, (b) the F1, (c) precision, and (d) the AP results of GFP *C. elegans* detection in YOLOv5.

6. YOLOv7

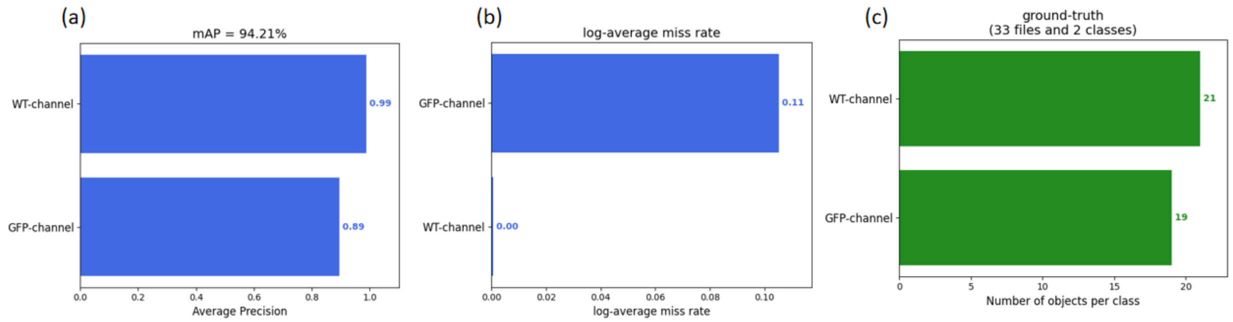

Figure S16. (a) The mAP, (b) the log-average miss rate, and (c) the ground-truth results in YOLOv7.

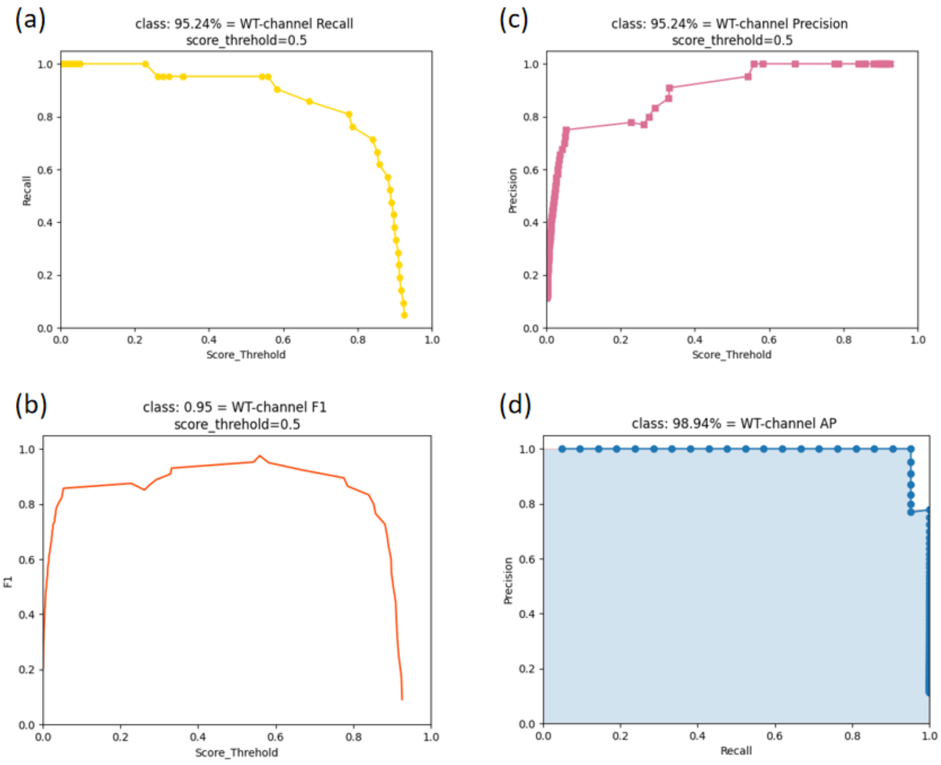

Figure S17. (a) The recall, (b) the F1 score, (c) the precision, and (d) the AP results of WT *C. elegans* detection in YOLOv7.

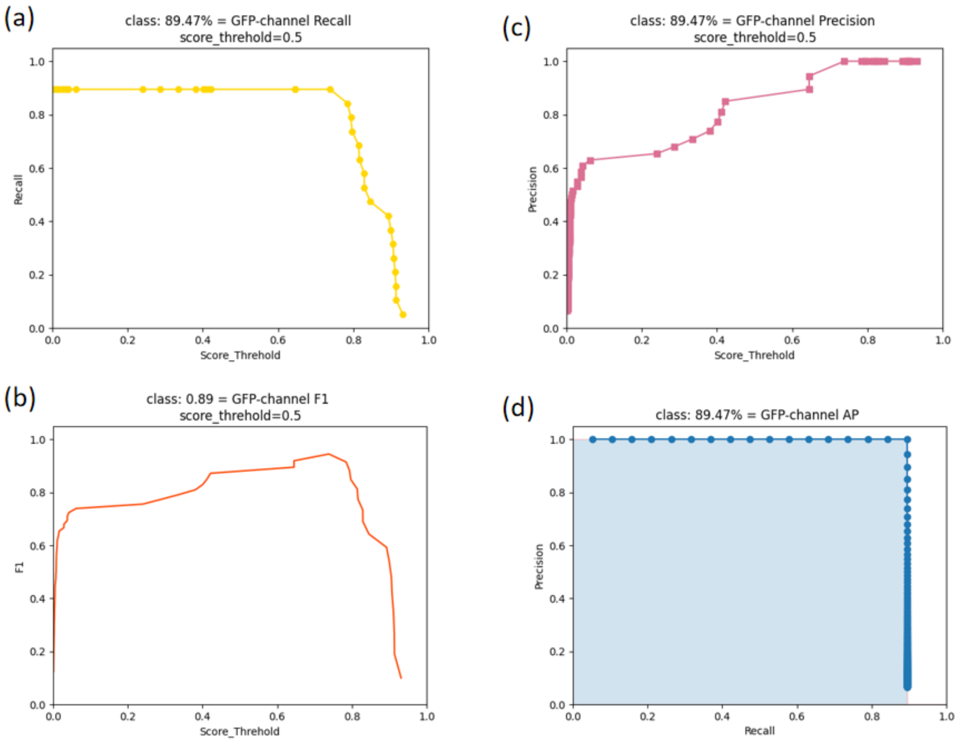

Figure S18. (a) The recall, (b) the F1 score, (c) the precision, and (d) the AP results of GFP *C. elegans* detection in YOLOv7.
